# Effect of cow-calf contact on cow motivation to reunite with their calf

**DOI:** 10.1038/s41598-020-70927-w

**Published:** 2020-08-28

**Authors:** Margret L. Wenker, Eddie A. M. Bokkers, Benjamin Lecorps, Marina A. G. von Keyserlingk, Cornelis G. van Reenen, Cynthia M. Verwer, Daniel M. Weary

**Affiliations:** 1 Animal Welfare Program, Faculty of Land and Food Systems, 2357 Main Mall, University of British Columbia, Vancouver, BC V6T 1Z6 Canada; 2 Animal Production Systems Group, Wageningen University and Research, PO Box 338, 6700 AH Wageningen, The Netherlands; 3 Livestock Research, Wageningen University and Research, PO Box 338, 6700 AH Wageningen, The Netherlands; 4 Louis Bolk Institute, Kosterijland 3-5, 3981 AJ Bunnik, The Netherlands

**Keywords:** Animal behaviour, Motivation

## Abstract

Early cow-calf separation prevents much of cows’ natural maternal behaviour. Early separation is thought to prevent the development of a cow-calf bond. To assess this bond, we measured motivation of dairy cows to reunite with their calf. To vary the degree of bonding, some cows were allowed continued contact with their calf and others were separated from their calf soon after birth, following standard practice on most farms. Among cows allowed continued contact, some were able to suckle their calf and others were prevented from suckling (by covering the cow’s udder with an udder net). Cows were habituated to the weighted-gate apparatus before calving by daily training with the (un-weighted) gate. After calving, cow willingness to use the gate was assessed by determining if she would push open the gate to access to her own calf. Testing occurred once daily, with weight on the gate gradually increased. After passing through the gate, the dam’s calf-directed behaviour was recorded. Suckled cows pushed a greater maximum weight (45.8 ± 7.8 kg) than separated cows (21.6 ± 6.7 kg) and non-suckled cows (24.3 ± 4.5 kg), with no differences between separated and non-suckled cows. Once reunited, latency to make nose contact and duration of licking did not differ between treatments. We conclude that motivation for calf contact is greater for cows that are suckled.

## Introduction

Maternal behaviour of cows can be expressed through licking, nursing, attentiveness and proximity towards the young, and protecting it from potential threats^1,2^. On most dairy farms the cow’s ability to express maternal behaviour is limited, as standard practice is to remove the calf within a few hours after birth^3^, but cows still perform maternal behaviour when given the opportunity^2,4,5^. To date no work has assessed to what extent dairy cows are still motivated to reunite and interact with their young. An essential component of maternal behaviour is nursing the calf^1^, but there is some evidence that the mother-young bond may establish in the absence of suckling: cow-calf pairs spent similar time in proximity regardless of the cows’ ability to suckle her calf^6^. It is unknown to what extent the maternal motivation to reunite with the calf is affected by suckling.

Motivation tests can be used to evaluate the value animals attribute to an experience or resource^7^. Once an animal has learned to perform a task, the effort (price) for each access can be increased^8,9^. Pushing a weighted door to gain access to a reward is one method to assess motivation^10,11^. This technique has been used to assess the importance of a nest box in chickens^12^, a water bath in farmed mink^13^, pasture access^14^ and access to an automated brush^15^ in dairy cattle. The more weight an animal pushes, the stronger the motivation to access that particular resource^16^.

The aim of this study was to assess the motivation of dairy cows with different levels of cow-calf contact to reunite with their calf. We hypothesised that cows routinely kept with their calf would be more motivated than control cows that has been separated from their calf soon after birth; previous work has shown that early separation greatly diminishes the bond between cow and calf^17^. Following Johnsen et al.^6^, who found no effect of suckling on the cow-calf bond, we hypothesised that among cows kept with their calf, the ability to nurse would not affect cow motivation.

## Results

### Weight pushed

**Figure 1:**
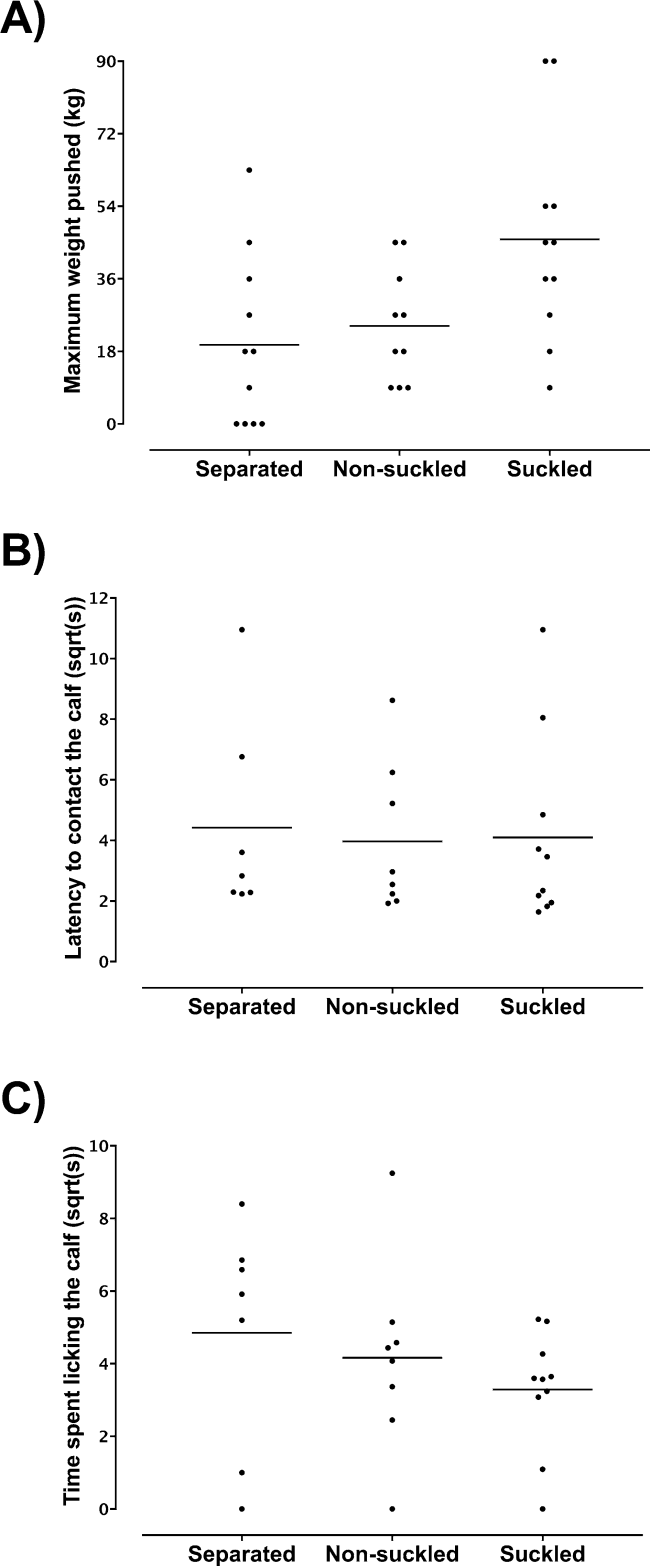
Cow motivation to reunite with their calf, and behaviours expressed upon reuniting, in relation to treatment; nightly cow-calf contact was allowed for non-suckled and suckled cows, but separated cows only spent time with their calf during the test. Results are shown separately for maximum weight (kg) pushed by dairy cows to reunite with their own calf (**A**), and for those cases in which the cow successfully opened the gate, latency (sqrt(s)) to contact the calf (**B**) and time (sqrt(s)) spent licking the calf (**C**). Values for each calf are shown separately (as black circles), with median values shown as a solid line. (**A**) Shows 11 cows in the separated treatment, versus 10 cows in the non-suckled and 11 in the suckled treatment. One of the four cows in the separated treatment that failed to open the weighted gate in test phase (i.e. appearing as 0 kg pushed in the plot), also failed to open the gate during the second training phase and on this basis was excluded from our statistical analysis. Measures of how cows interacted with their calves during the test session [i.e. latency to approach the calf (**B**) and time spent licking the calf (**C**)] are only available for the cows that actually opened the weighted gate during the test session, resulting in a sample size of 7 for the separated treatment, versus 10 cows in the non-suckled and 11 in the suckled treatment.

The mean maximum weight pushed ± SEM (in kg) did not differ between separated cows (21.6 ± 6.7) and non-suckled cows (24.3 ± 4.5; p = 0.78); whereas, suckled cows pushed a greater maximum weight (45.8 ± 7.8) than separated cows (p = 0.03) and non-suckled cows (p = 0.01) (Fig. 1).

### Calf-directed behaviour at reunion

The latency for cows to make nose contact (median [95% CI] in s) did not differ between separated cows (10.5 [5.0–120.0]), non-suckled cows (10.3 [3.7–78.0]), or suckled cows (22.4 [2.7–64.6]; p = 0.97). In addition, no difference was found in the duration of licking (median [95% CI] in s) between separated cows (43.4 [0.0–70.5]), non-suckled cows (18.1 [0.0–85.5]) or suckled cows (12.8 [1.2–27.3]; p = 0.30).

## Discussion

Our results showed that suckled cows were more motivated to reunite with their calf than were separated cows and cows that were not separated but also not suckled. Previous work suggested that a strong cow-calf bond can be established even in the absence of suckling^6^, but the results of the present study indicate that suckling increases the cow’s motivation to reunite with her calf. This increased motivation may be due to a stronger bond between the cow and calf. One of the hormones involved in mother-young bonding is oxytocin^18^, and this is known to be increased in suckled cows compared to non-suckled cows^19^. Oxytocin and the endogenous opioids released during suckling have a rewarding effect^20^, and suckling has been considered as one of the most hedonic maternal activities^21^.

In the present study, motivation to reunite with the calf did not differ between separated and non-suckled cows. Close contact with the calf in the first few hours after birth is considered essential to establish a maternal bond, and provides the dam with olfactory and gustatory input to recognize on her offspring^22^. It has been suggested that as little as 5 min of contact directly after birth is enough to establish a mother-young bond that could withstand 12 h of separation^23^, although interactions with the young are considered important to maintain high maternal motivation^24^.

Cows in all three treatments expressed similar amounts of calf-directed behaviour once reunited, preventing any meaningful conclusions based upon these measures.

There are a number of limitations to the current study. Previous studies using a similar push gate showed that dairy cows are highly motivated to access pasture^14^ and an automated brush^15^, but differences in the way these gates were installed prevents meaningful comparison in the weights pushed across studies. We suggest future studies directly monitor the force applied to the gate, rather than the weights lifted as reported here, as the force applied to the gate will be more easily compared across studies. The current study used a between-subject design, such that each cow was allocated to a single treatment. Cows varied considerably in their responses, both in the weight pushed and the behaviours shown when reunited with their calves, and this individual variation potentially obscured treatment differences with our design. In addition, we had expected that the separation treatment would act as a type of null control, with cows showing little or no motivation to access their calf. Our finding that some of the separated cows worked to access their calf, and engaged in a considerable degree of calf directed behaviour upon being reunited, was an unexpected and important finding, but also diminishes our ability to use this treatment as a type of null control. Future work should consider using larger samples, or within-subject designs comparing each cow’s motivation to access the calf versus other valuable resources (such as pasture or food^14^).

## Methods

### Animals and treatments

Holstein cows that recently gave birth (*n* = 34; mean parity 3 ± 0.3 lactations), were assigned randomly without replacement to one of the three treatments within each block of three successive calvings. The treatments were: (i) separated from their calf within 2 h after birth and allowed no contact outside of testing sessions (*n* = 11, separated), (ii) allowed to spend nights with their calf but fitted with an udder net to prevent suckling (*n* = 11, non-suckled), or (iii) allowed to spend nights with their calf and to be suckled (*n* = 12, suckled). All cows were milked twice a day. Nightly cow-calf contact was allowed from approximately 18:30 h until 06:30 h. See electronic supplementary material S1 for more details. All methods were carried out in accordance with relevant guidelines and regulations.

### Motivation test

A one-way push gate (adapted from^14^) in the test pen allowed access to the calf. The push gate could be opened by physically pushing against a plate attached to the gate. See electronic supplementary material S2.

#### Training

The training period was divided into two phases. In the first phase, 3 weeks before expected calving date, cows were trained to open the push gate to access fresh feed (6 repetitions per day). The second training phase started 2–4 days after calving; cows were trained to reunite with their calf via the push gate (1 repetition per day). During this stage, fresh feed was freely available inside the test pen before accessing the push gate; thus cows learned that opening the gate would provide access to the calf only and not to fresh feed. To be included in the experiment, cows had to push open the gate successfully in both phases. See electronic supplementary material S1 for more details.

#### Testing

The bond between cow and calf develops rapidly after birth^17^, such that responses to separation are much stronger after just 4 days of continued contact (relative to separation in the hours after birth;^25^). For this reason, we started testing 5–8 days after calving. Cows were tested individually, after the afternoon milking (i.e. 17:00–18:30 h); the order in which cows were tested was randomised.

The test started with the gate closed but with no weight attached to the pulley system. Weight was then increased every day upon success: initially to 2.3 kg, then to 9 kg followed by 9 kg increments each day. If a cow was unsuccessful in pushing the gate open, she was retested at that same weight for the following 2 days. Testing ended when a cow failed to open the gate for three consecutive days or if she pushed the maximum weight of 90 kg. After each test session, cow and calf were returned to their corresponding pen depending upon treatment. Maximum weight pushed for each cow was recorded, and a digital camera was used to record calf-directed behaviour after reunion (i.e. latency to make nose contact, duration of licking).

### Statistical analysis

Three cows were excluded from the analyses: one cow from the separated group (she did not meet the learning criterion in the second training phase; all other cows met this criterion), one cow in the non-suckled group (her calf learned to evade the udder net and started suckling half way the experiment), and one cow in the suckled group (her calf was born premature and never learned to suckle), resulting in a final sample of 31 cows (10 in both the separated and non-suckled groups, and 11 in the suckled group). All statistical analyses were performed using SAS (version 9.4, SAS Institute, Institute Inc., Cary, NC), treating cow as the experimental unit. Residuals were visually assessed for normality. Significance was declared at *P* < 0.05.

#### Weight pushed

To test the effect of treatment on the maximum amount of weight pushed, the least significant difference mean comparison test in PROC GLM procedure was used. Residuals were inspected for normality and outliers.

#### Calf-directed behaviour at reunion

Behavioural observations of 7 separated cows, 10 non-suckled cows, and 11 suckled cows were included; 3 separated cows did not open the gate to reunite with their calf during the test. As cows that pushed the gate successfully had more test sessions than the unsuccessful ones, latency to make nose contact and duration of licking were averaged for each cow. None of the recorded behaviours were normally distributed, so treatment differences were analysed using a Wilcoxon rank-sum test.

### Guidelines followed for the care and use of animals

All procedures involving animal handling and testing were approved by the University of British Columbia’s Animal Care Committee (#A15-0082).

## Supplementary information

Supplementary Information 1.

Supplementary Information 2.

Supplementary Information 3.

Supplementary Information 4.

Supplementary Information 5.

## Data Availability

The code (S3) and datasets (S4 and S5) supporting this article have been uploaded as part of the electronic supplementary material.
