# Supplementary material for: Effect of cow-calf contact on cow motivation to reunite with their calf
Source: Sci Rep. 2020 Aug 28;10:14233. doi: 10.1038/s41598-020-70927-w (PMC7455555; doi:10.1038/s41598-020-70927-w)
Supplement: Supplementary file 1 — Supplementary Information 1. [file 41598_2020_70927_MOESM1_ESM.docx]

**Electronic Supplementary Material S1 – Detailed Material & Methods for:**

**Effect of cow-calf contact on cow motivation to reunite with their calf**

Margret L. Wenker, Eddie A.M. Bokkers, Benjamin Lecorps, Marina A.G. von Keyserlingk, Cornelis G. van Reenen, Cynthia M. Verwer, and Daniel M. Weary

Corresponding author email: dan.weary@ubc.ca

***Animals & Experimental setup***

This research was conducted at the UBC Dairy Research and Education Centre in Agassiz (Canada) from June 2018 to November 2018. The experimental cows had an average body weight of 676 ± 14 kg. The treatments only differed in the level of cow-calf contact that was allowed during the trial. Non-suckled dams were fitted an udder net (model Nr 88439503, DeLaval, Tumba, Sweden) to cover the udder directly following parturition. During the day (between 06:30 h and 17:00 h), cows were moved to a separate pen without visual contact with the calves (see section Housing). All cows were milked twice a day at approximately 07:00 h and 17:00 h in a double 12 stall parallel milking parlour. Both non-suckled and suckled cow-calf pairs were reunited every day after afternoon milking. Cows were fed a total mixed ration (TMR; shown as percent of dry matter, consisting of 90.9 % alfalfa hay, 80.8 % grass, 29.8 % corn silage, and 89.2 % concentrates) twice a day at approximately 08:30 h and 16:00 h. All calves were provided *ad libitum* fresh milk twice a day at approximately 07:00 h and 16:00 h using a portable milk bar (Milk Bar 10 calf feeder, Coburn, USA). All calves were trained to use the milk bar by guiding them to the nipple in the first week of life. In the calf creep area (see section Housing) *ad libitum* water, hay, and TMR was provided.

***Housing***

Two identical free-stall pens were used to house the cows containing each 12 lying stalls deep bedded with sand (115 cm wide x 205 cm long), 6 electronic feeding bins, and 1 electronic water bin (Insentec, Marknesse, the Netherlands) (Figure 1A). During the day (between 06:30 h and 17:00 h) all experimental cows were housed in the same pen (i.e. ‘home pen’), the group size never exceeded 12 animals. All experimental calves were kept in a sawdust-bedded creep area (8.5 m long x 3.0 m wide) (Figure 1A). At night (between 18:30 h and 06:30 h) separated cows stayed in the home pen and their calves stayed in the calf creep area. Non-suckled and suckled cow-calf pairs were moved to an adjacent pen (i.e. ‘contact pen’). Plywood plates (160 cm high) were placed between the two free-stall pens to prevent visual contact.

***Calving management***

Calving took place in individual indoor maternity pens bedded with sawdust. Separated calves were removed from the dam and placed in the calf creep area within 2 h after birth (median [minimum, maximum] in min: 15 [5, 110]). After parturition separated cows spent on average 15.8 h (± 0.6) in the maternity pen before they returned to the home pen. Non-suckled and suckled calves stayed with the cow in the maternity pen for on average 32.0 h (± 0.6) and were moved to the calf creep in the morning. All fresh cows were moved to the home pen after morning milking. All calves were bottle fed 4 L of colostrum within 6 h after birth. In the maternity pen non-suckled and suckled calves were also bottle fed 4 L fresh milk twice a day until they moved to the calf creep area.

***Push gate apparatus***

In the test pen (Figure 1B) a one-way push gate (adapted from (1)) was installed and a pulley was used to load the push gate with weights (see electronic supplementary material (S2) for a video). A plywood plate (0.6 x 0.9 m) was attached to the gate for cows to place their head against when pushing the gate. The sides of the entrance to the gate were both covered with plywood boards (1.2 m x 2.4 m) to prevent cows from being visually distracted once approaching the gate.

***Training***

In the first training phase dry cows were trained on a daily basis to go through the push gate (on average 6 repetitions) to access a bucket with fresh TMR (for 30 s; Figure 1B). Training started with the gate completely open (i.e. gate angle 45°) for at least 2 repetitions to get familiar with the task, then the gate was closed progressively by 15° until it was fully closed with 2.3 kg attached (Table 1). A cow had to complete each step before she was allowed to progress to the next one. If a cow failed, the gate was returned to its previous position to repeat this learning step again. Training was considered successful when a cow opened the fully closed gate with 2.3 kg for 3 consecutive repetitions. Each daily training session lasted approximately 15 min per cow. All cows passed this training phase within 5 d.

The second training phase after calving lasted 3-5 d; cows were trained to reunite with their calf via the push gate. After afternoon milking, all experimental cows were brought to a waiting pen adjacent to the test pen (Figure 1C). From there, cows were individually brought to the test pen. The calf was fitted a rope halter inside the calf creep area and was brought into the alley behind the push gate. Here, it was restrained (rope of 1 m) at approximately 2 m from the push gate. In this training phase cows had only one repetition each day for each of the 3 learning steps (Table 1). For the last learning step TMR was put inside the test pen on the opposite side of the push gate (Figure C); thus, cows could choose to go to the left to the red bucket with fresh TMR or to push open the gate and thus access her calf. Once they opened the push gate, cows were able to spend 2 min with their calf after which they were returned to either the home pen or contact pen depending on the treatment. For non-suckled and suckled cows the three training days were also used to get them familiar with the routine of having cow-calf contact overnight and being separated during the day. To be included in the study, cows had to pass the push gate voluntarily in learning step III (Table 1) within the maximum set time of 5 min after entering the test pen.

***Behavioural observations***

During the test a digital camera (Sony Handycam HDR-CX560) fixed on a tripod was placed in the alley at 7 m away from the calf to record latency to make nose contact and duration of licking. Latency to make nose contact with the calf was defined as time (in s) from the cow moving her shoulder through the gate until her nose contacted any part of the calf’s body. Duration of licking the calf was defined as time (in s) that the cow licked any part of the calf’s body.

**References**

1. von Keyserlingk MAG, Amorim Cestari A, Franks B, Fregonesi JA, Weary DM. Dairy cows value access to pasture as highly as fresh feed. Sci Rep; 7:44953.

**Table 1.** Details on the training to operate the push gate.

| **Training phase** | **Learning step** | **Gate angle** | **Reward** |
| --- | --- | --- | --- |
| First training phase | Step 1^a^ | 45° | 30 s to eat TMR |
|  | Step 2 | 30° | 30 s to eat TMR |
|  | Step 3 | 15° | 30 s to eat TMR |
|  | Step 4 | 0° | 30 s to eat TMR |
|  | Step 5 | 0° + 2.3 kg | 30 s to eat TMR |
|  |  |  |  |
| Second training phase | Step I | 30° | 2 min with calf |
|  | Step II | 15° | 2 min with calf |
|  | Step III^b^ | 15° | 2 min with calf |

^a^ All cows made at least 2 repetitions in this step before we proceeded to the next step.

^b^ In this training step TMR was present in the test pen.

**A)
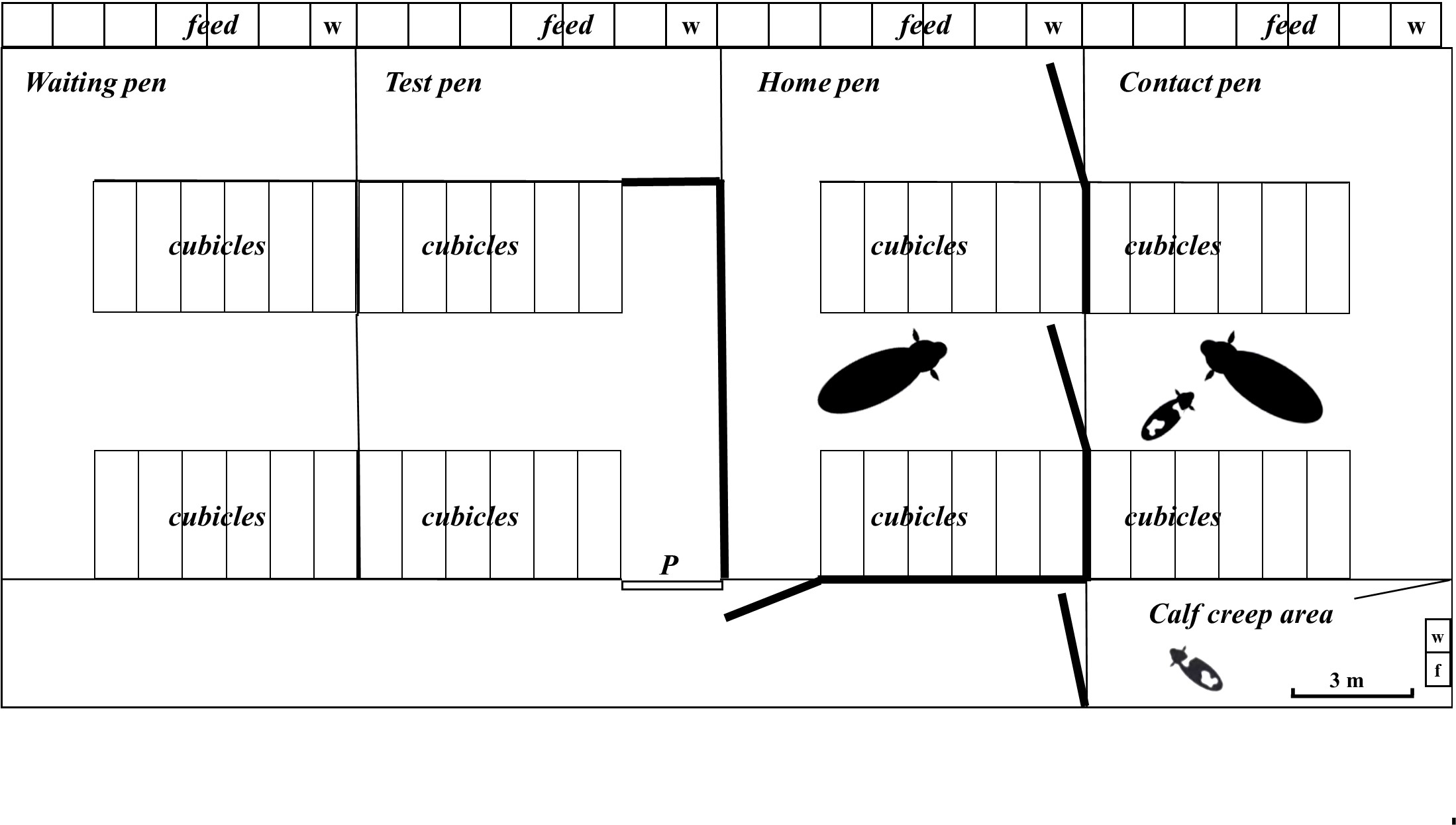
**

**B)**

**
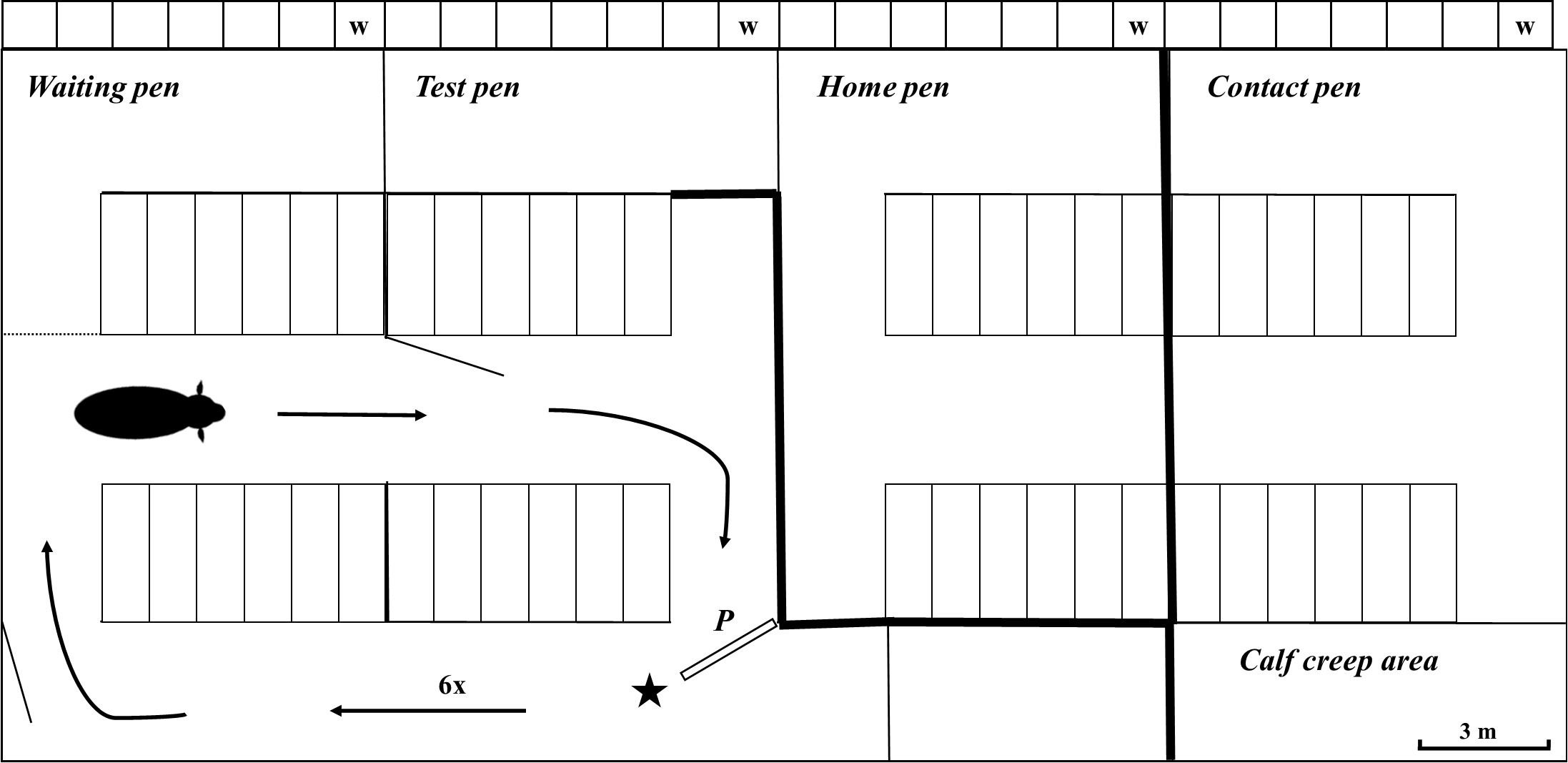
**

**C)** **
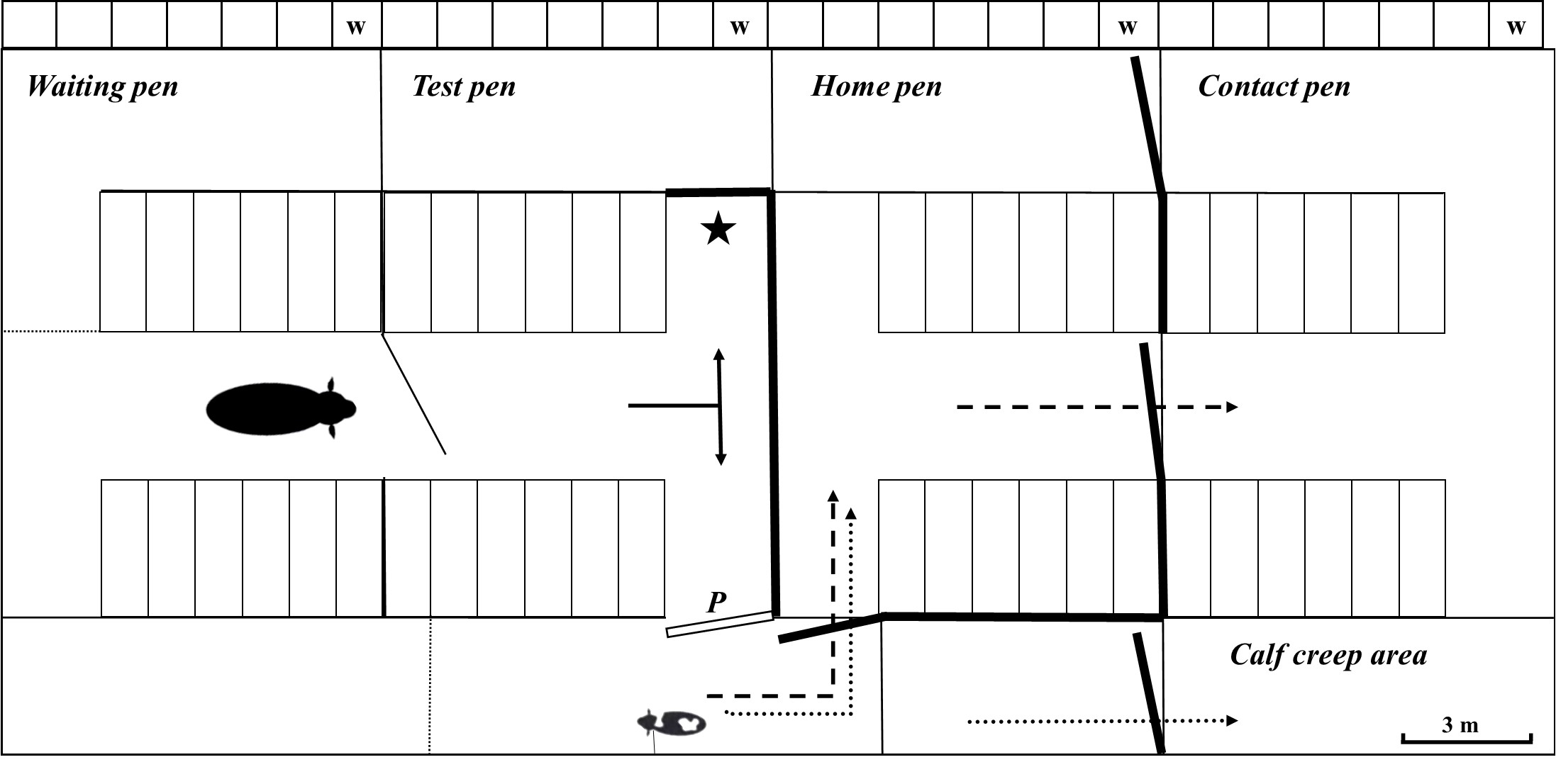
Figure 1**. Experimental set-up. **A)** During the day (between 06:30 h and 17:00 h) cows were kept as one dynamic group in the ‘home pen’ (9.5 m x 11.5 m), all calves were housed in the sawdust-bedded calf creep area (9.5 m x 3 m). At night (between 18:30 h and 06:30 h), non-suckled and suckled cow-calf pairs were moved to the ‘contact pen’. Separated animals stayed in the home pen. **B)** In the first training phase cows were trained to open the push gate apparatus. A red bucket with fresh TMR was used as reward, and was placed in the alley behind the push gate. **C)** In the second training phase cows were trained to reunite with their calf. The calf was tethered in the alley behind the push gate and TMR was present in the test pen (learning step III). After passing the second training phase, the test started the following day. After training and testing separated cows were returned to the home pen and their calves to the calf creep area; non-suckled and suckled cow-calf pairs would be brought to the contact pen. ★ = TMR, ***P*** = push gate, w = water, f = feed. **▬▬** = plywood, ⬝⬝⬝⬝⬝ = route separated cow-calf pairs to their pens, **- - - -** = route non-suckled and suckled cow-calf pairs to contact pen.
